# Passive Acoustic Monitoring and Deep Learning Reveal a Lag From Rainfall to Gibbon Song Across a Mosaic Forest Landscape

**DOI:** 10.1002/ece3.73717

**Published:** 2026-05-27

**Authors:** Alasdair F. Owens, Erik Estrada, Kimberley J. Hockings, Muhammed Ali Imron, Mariaty, Manmohan D. Sharma, Siti Maimunah, Tommy J. Travers‐Cook, Frank J. F. Van Veen, Wendy M. Erb

**Affiliations:** ^1^ Centre for Ecology and Conservation, Faculty of Environment, Science and Economy University of Exeter Penryn UK; ^2^ Yayasan Borneo Nature Indonesia Palangka Raya Indonesia; ^3^ Faculty of Forestry Universitas Gadjah Mada Yogyakarta Indonesia; ^4^ Fakultas Kehutanan Dan Pertanian Universitas Muhammadiyah Palangkaraya Palangka Raya Indonesia; ^5^ Department of Earth and Environmental Science, Faculty of Environment, Science and Economy University of Exeter Penryn UK; ^6^ Fakultas Kehutanan Instiper Yogyakarta Yogyakarta Indonesia; ^7^ Department of Zoology University of British Columbia Vancouver British Columbia Canada; ^8^ Department of Microbiology and Immunity University of British Columbia Vancouver British Columbia Canada; ^9^ Cornell K. Lisa Yang Center for Conservation Bioacoustics Cornell University Ithaca New York USA; ^10^ Cornell Lab of Ornithology Cornell University Ithaca New York USA

**Keywords:** automated detection, bioacoustics, CNN, endangered species, primates, rainfall, vocal behaviour

## Abstract

Understanding the fundamental ecology of endangered species is essential for effective conservation, yet this remains challenging for elusive species inhabiting tropical forests. For the endangered Bornean white‐bearded gibbon ( *Hylobates albibarbis* ), much of the available ecological information derives from peat swamp forests, while comparatively little is known from other forest types that make up a large part of its range. Passive acoustic monitoring provides an opportunity to address this gap, enabling the study of species' vocal behaviour over larger spatial and temporal scales than previously possible. We deployed eight autonomous recording units across three forest types in Central Kalimantan, Indonesia, collecting 23,244 h of acoustic data over 18 months. A pretrained deep learning detector was applied to identify great calls, performed by female gibbons as part of morning duets and used as a key indicator for comparing population density. We identified 83,956 great calls and examined how daily calling activity varied across habitats and in response to seasonal rainfall. Daily calling activity differed significantly among forest types, consistent with expected differences in gibbon population density. Significant temporal variation in calling behaviour was observed consistently across habitats. We documented negative short‐term and positive long‐term effects of rainfall on calling activity. Daily calling activity peaked 51–52 days following rainfall events, with effect sizes increasing with rainfall dose, suggesting that calling activity reflects a lagged phenological fruiting response to seasonal rainfall. Our findings highlight the importance of accounting for variable vocalisation rates in acoustic monitoring, particularly when evaluating the additive effects of anthropogenic disturbance and climate change on species' behaviour and ecology. We emphasise the value of incorporating spatial data to strengthen ecological inferences from acoustic datasets, and demonstrate the power of deep learning for long‐term monitoring of species' vocal behaviour, providing deeper ecological understanding across increasingly broad spatiotemporal scales.

## Graphical abstract

We deployed passive acoustic monitoring across three forest types in Central Kalimantan, Indonesia, and applied a pretrained deep learning automated detector to identify 83,956 Bornean white‐bearded gibbon ( *Hylobates albibarbis* ) great call vocalisations over 18 months. Daily calling activity differed significantly among forest types and showed significant temporal variation, peaking approximately 50 days following rainfall and increasing with rainfall dose. Our findings demonstrate the power of deep learning for large‐scale, long‐term monitoring of species' vocal behaviour, while highlighting the need to account for variable vocalisation rates when evaluating the additive effects of habitat loss and climate change on species' behaviour and ecology.

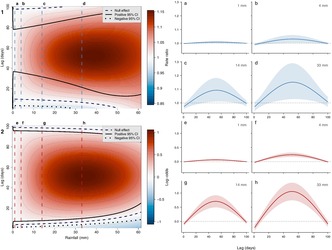

## Abstrak

Memahami ekologi dasar spesies terancam punah merupakan kunci bagi upaya konservasi yang efektif. Namun, hal ini menjadi tantangan besar, terutama bagi spesies yang sulit diamati di hutan tropis. Pada owa jenggot putih Kalimantan (*Hylobates albibarbis*), sebagian besar informasi ekologi yang tersedia masih terbatas pada hutan rawa gambut, sementara pengetahuan dari tipe hutan lain, yang justru mencakup sebagian besar wilayah jelajahnya, masih minim. Penggunaan teknologi *passive acoustic monitoring* menawarkan peluang untuk mengatasi kesenjangan ini, dengan memungkinkan studi perilaku vokal spesies pada skala ruang dan waktu yang lebih luas dibandingkan metode konvensional. Kami menempatkan delapan unit perekam suara otonom di tiga tipe hutan di Kalimantan Tengah, Indonesia, dan mengumpulkan total 23.244 jam data akustik selama 18 bulan. Detektor *deep learning* pralatih digunakan untuk mengidentifikasi *great calls*, yaitu vokalisasi khas yang dilakukan oleh owa betina sebagai bagian dari duet pagi. Vokalisasi ini digunakan sebagai indikator utama dalam estimasi kepadatan populasi. Secara keseluruhan, kami mengidentifikasi 83.956 *great calls* dan menganalisis variasi aktivitas panggilan harian antar habitat serta responsnya terhadap curah hujan musiman. Aktivitas panggilan harian menunjukkan perbedaan yang signifikan antar tipe hutan, sejalan dengan variasi perkiraan kepadatan populasi owa. Selain itu, terdapat variasi temporal yang jelas dalam perilaku panggilan, meskipun pola ini relatif konsisten di seluruh habitat. Kami menemukan adanya efek negatif jangka pendek serta efek positif jangka panjang dari curah hujan terhadap aktivitas panggilan. Aktivitas panggilan mencapai puncaknya 51–52 hari setelah periode curah hujan, dengan besaran efek yang meningkat seiring intensitas hujan. Pola ini konsisten dengan aktivitas panggilan yang mengikuti respons fenologi pembuahan yang tertunda terhadap curah hujan musiman. Temuan ini menegaskan pentingnya mempertimbangkan variasi laju vokalisasi dalam pemantauan akustik, khususnya dalam mengevaluasi dampak kumulatif gangguan antropogenik dan perubahan iklim terhadap perilaku serta ekologi spesies. Kami juga menekankan pentingnya integrasi data spasial untuk memperkuat inferensi ekologi dari *dataset* akustik. Lebih lanjut lagi, hasil penelitian menunjukkan potensi besar pendekatan *deep learning* dalam pemantauan jangka panjang dan berskala luas terhadap perilaku vokal, sehingga dapat memperluas pemahaman ekologi spesies pada dimensi ruang dan waktu yang lebih komprehensif.

## 1 Introduction

Effective conservation action requires an understanding of the fundamental ecology of threatened species, including distribution, habitat use, and the limiting factors on their population density (Rushton et al. 2004). However, obtaining such information is particularly challenging for animals inhabiting tropical rainforests, which are among the most biodiverse and threatened biomes on Earth. This is due to limitations on direct observation caused by dense foliage, human‐avoidance behaviour, and the inaccessibility of habitats to researchers (Zwerts et al. 2021). The gibbons (family Hylobatidae) of Southeast Asia are a taxonomic group that exemplify these challenges. Despite 19 of the 20 species being classified as Endangered or Critically Endangered by the IUCN Red List of Threatened Species, key ecological knowledge remains limited (IUCN 2026). For example, while the boundaries of species' ranges may be well known, occupancy and population density distributions within those ranges, and how these may be affected by habitat variation, remain largely unknown (Cheyne et al. 2016; Geissmann 2007). Furthermore, where detailed behavioural knowledge from direct observation does exist, it is typically restricted to small geographic areas and a limited number of habituated individuals; meanwhile, there is a notable lack of data on gibbons outside of protected areas (Cheyne et al. 2023).

The endangered Bornean white‐bearded gibbon ( *Hylobates albibarbis* ) is endemic to southern Borneo, occurring within Indonesia's Central Kalimantan and West Kalimantan provinces, south of the Kapuas River and west of the Barito River (Marshall et al. 2020). Variation in soil types and elevation has given rise to a range of forest types in this area, with distinctly different tree species compositions (Anirudh et al. 2025). This botanical variation among forest types influences the distribution of gibbons across their range; for example, greater canopy height and cover are associated with higher population densities (Hamard et al. 2010). However, the majority of research on *H. albibarbis* ecology and behaviour has been conducted in only a handful of peat swamp forest locations. While an estimated 50% of *H. albibarbis'* total population inhabits peat swamp forests, comparatively little is known about populations inhabiting the other forest types that comprise the remainder of the species' range (Cheyne et al. 2016).

Most gibbon species perform elaborate long‐range vocalisations in the form of sex‐specific songs that are coordinated as morning duets by territorial mated pairs (Geissmann 2002). These duets typically consist of introductory, interlude, and great call sequences, with the latter being the most stereotyped and easily identifiable component (Geissmann 2002). The great call sequence, comprising the female great call, often followed by a male coda, serves as a key indicator for comparing population density, as its presence indicates a mated pair (Gilhooly et al. 2015). These duets serve multiple functions, including mediating intergroup spacing, maintaining the pair‐bond, mate defence, and advertising attributes of the individual or pair (Cowlishaw 1992; Geissmann and Orgeldinger 2000; Mitani 1985). Studies of gibbon songs have been used to identify species (Cheyne et al. 2024), determine phylogenetic relationships (Thinh et al. 2011), estimate population density (Cheyne et al. 2016), assess spatial distribution (Okuda et al. 2022), and identify individuals (Clink et al. 2017). Recent advances in passive acoustic monitoring (PAM) and deep learning have further expanded the scope of such research by improving the efficiency of collecting and analysing acoustic data while enabling research across larger spatial and temporal scales than previously possible (Stowell 2022; Wich and Piel 2021).

The deployment of PAM arrays across diverse forest types provides the opportunity to infer differences in gibbon population densities among habitats from the relative calling activity detected in recordings over prolonged periods. Gibbon groups occupy fixed territories with strong site fidelity, so population densities within a given area remain relatively stable over time (Cheyne et al. 2019). Consequently, prolonged differences in calling activity between habitats could reflect genuine differences in population density rather than short‐term behavioural variation. It should, however, be noted that the relationship between calling activity and population density may not be linear, as gibbon singing has been shown to be density dependent, wherein gibbon groups sing less frequently in areas with fewer neighbouring groups (Brockelman and Srikosamatara 1993; Yin et al. 2016). Further, consistent differences in call rates may reflect variation in individual or group‐level calling behaviour that does not necessarily correspond to density (Clink et al. 2020); for instance, the number of great calls in a duet can be influenced by pair‐bond strength (Ma et al. 2022). While these relationships may complicate direct interpretation, meaningful differences in population densities among habitats would nonetheless be expected to manifest as amplified differences in relative calling activity.

Further, singing is energetically costly, and gibbons have been observed to produce shorter songs and call less frequently during periods of low food abundance (Cowlishaw 1996). As gibbons are primarily frugivorous, temporal variation in fruit availability is likely a key driver of variation in calling activity. The timing and magnitude of fruit availability can differ greatly among forest types, with some showing masting, whereby mass fruiting events in some years are interspersed by a varying number of lean years, whereas other types show either no or limited masting but may show varying degrees of intra‐annual variation in response to seasonal variation in rainfall (Brearley et al. 2007). In the latter case, the rainy season is typically associated with peaks in the abundance of animal‐dispersed pulpy fruit (van Schaik and Pfannes 2005), which could be expected to coincide with increased gibbon calling activity. Conversely, immediate rainfall generally reduces both the duration and probability of pairs engaging in song duets, possibly because rainy or windy conditions impair sound transmission, reducing the effectiveness of long‐range vocal communication, and/or because the increased energetic cost of overnight thermoregulation may cause gibbons to prioritise foraging over singing the following morning (Brockelman and Srikosamatara 1993; Cheyne 2008; Clink et al. 2020; Mitani 1985). A positive association between calling activity and seasonal rainfall would therefore be consistent with the hypothesis that resource availability is an important driver of gibbon singing behaviour, despite the short‐term suppressive effects of adverse weather.

Here, we use an 18‐month acoustic data set collected using a PAM array deployed in a mosaic lowland forest landscape in southern Borneo. We utilise a deep learning detector developed by Owens et al. (2024) to automatically identify *H. albibarbis* great calls. We investigate how daily *H. albibarbis* calling activity, defined here as both the number of great calls detected per day and the probability of a great call detection, varies across habitats and in response to seasonal rainfall. Specifically, we test the following hypotheses: (1) daily calling activity differs among habitat types, with higher calling activity in forest types with taller, more continuous canopy cover, reflecting expected differences in population density; (2) daily calling activity varies seasonally, with temporal trends differing among habitats, which may reflect the timing of peaks and troughs in resource abundance in different habitat types; (3) daily calling activity is positively associated with seasonal rainfall, despite the suppressive short‐term effects of rainfall on calling activity, which would suggest that resource availability is an important driver of singing behaviour.

## 2 Methods

### 2.1 Study Site

The long‐term acoustic dataset used in this study derives from the Mungku Baru Education and Research Forest (MBERF), a ~50 km^2^ area of tropical rainforest in Central Kalimantan Province, Indonesia (1°39′S 113°44′E). The region experiences a seasonal tropical climate with a pronounced rainy season typically occurring from November to April and a dry season from June to September (Brearley et al. 2007), though inter‐annual variation is considerable and prolonged dry spells can occur during El Niño years, as in 2019 (Yokelson et al. 2022).

**FIGURE 1:**
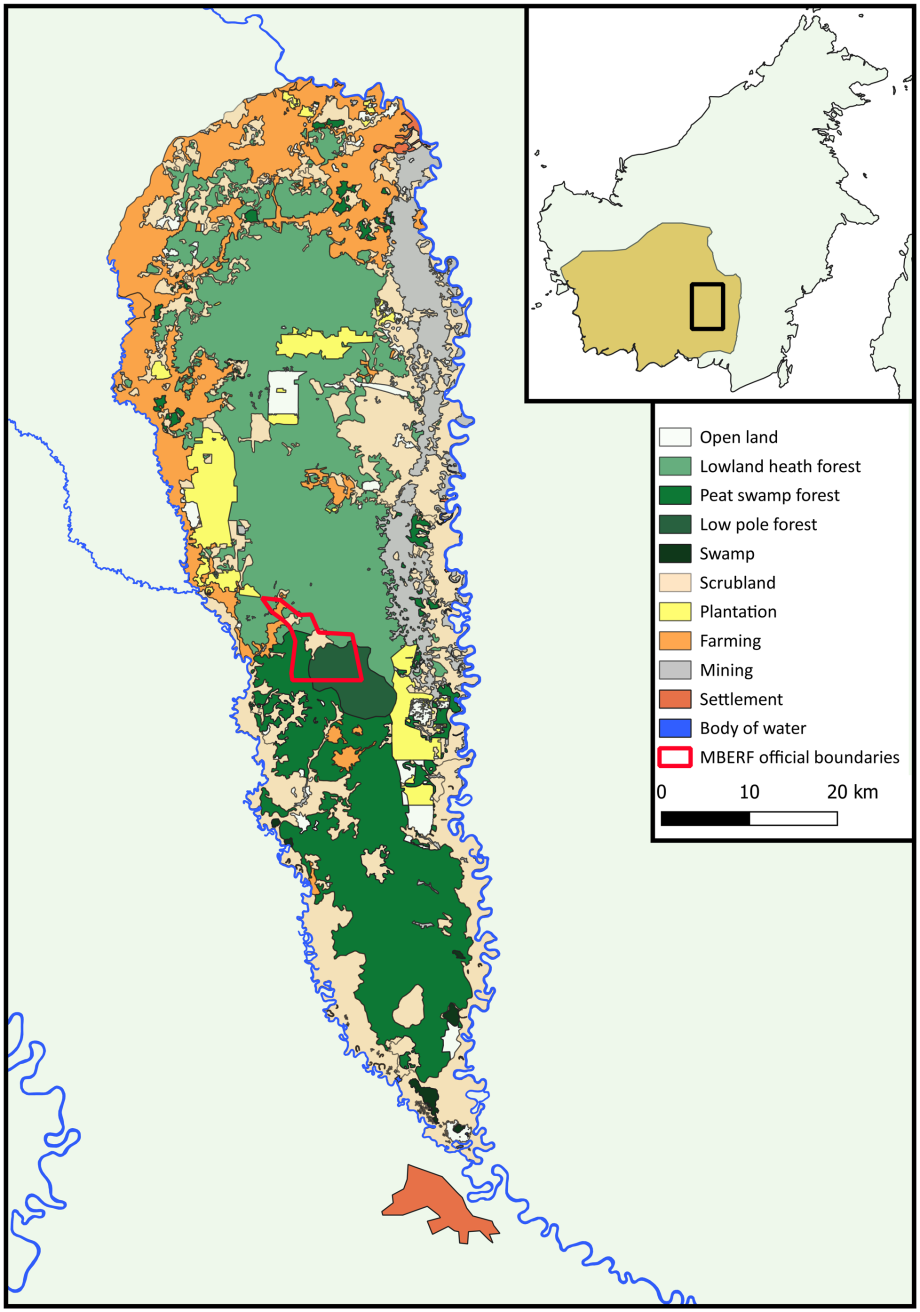
Map of the Rungan forest landscape, showing the official land designations and the location of the Mungku Baru Education and Research Forest (MBERF; General Directorate of Forestry Planning and Environmental Management, Indonesian Ministry of Forestry 2020). The inset shows the location of the Rungan forest landscape and the known range of *H. albibarbis* within Borneo (IUCN 2026).

The MBERF lies in the centre of the wider Rungan forest landscape, which spans approximately 1500 km^2^ between the Kahayan and Rungan rivers, north of the provincial capital of Palangka Raya (Figure 1). This landscape represents the largest area of continuous unprotected lowland rainforest remaining on the island of Borneo (Afitah and Purnama 2021) and is home to an estimated 4000 *H. albibarbis* individuals (Buckley et al. 2018), making the region critically important for the conservation of the species. Despite this, the forests here are under threat from the conversion of primary forest to oil palm and acacia plantations, expansion of coal mining concessions, gold mining in surrounding rivers, wildlife hunting, and forest fires. Ongoing wildlife monitoring has been recommended to strengthen the case for increased protection and fully realise the conservation potential of the Rungan forest landscape (Anirudh et al. 2025; Buckley et al. 2018).

The MBERF lies at low altitude (~60 m above sea level), with a gently undulating topography giving rise to a mosaic of different habitats, including three predominant forest types: ‘lowland heath’ (known locally as kerangas), ‘low pole peat swamp’ (low pole), and ‘mixed swamp’, with the latter representing a transitional habitat between the former two (Anirudh et al. 2025; Buckley et al. 2018). Compared to low pole, lowland heath has taller trees (average height ~18 m) and greater tree diversity (12.05–15.74 species/100 stems). Low pole, on the other hand, has an average tree height of 14.90 m, lower tree diversity (6.43 species/100 stems), and is characterised by a low, open canopy. Mixed swamp has the highest tree diversity (19.67 species/100 stems). As a transitional habitat, mixed swamp exhibits a gradient from tall continuous to low discontinuous canopy cover (Anirudh et al. 2025). The northern part of the MBERF also features an area that was impacted by a tornado event in 2006 and is characterised by windblown trees and dense regrowth, forming a low, discontinuous canopy.

### 2.2 Data Collection

**FIGURE 2:**
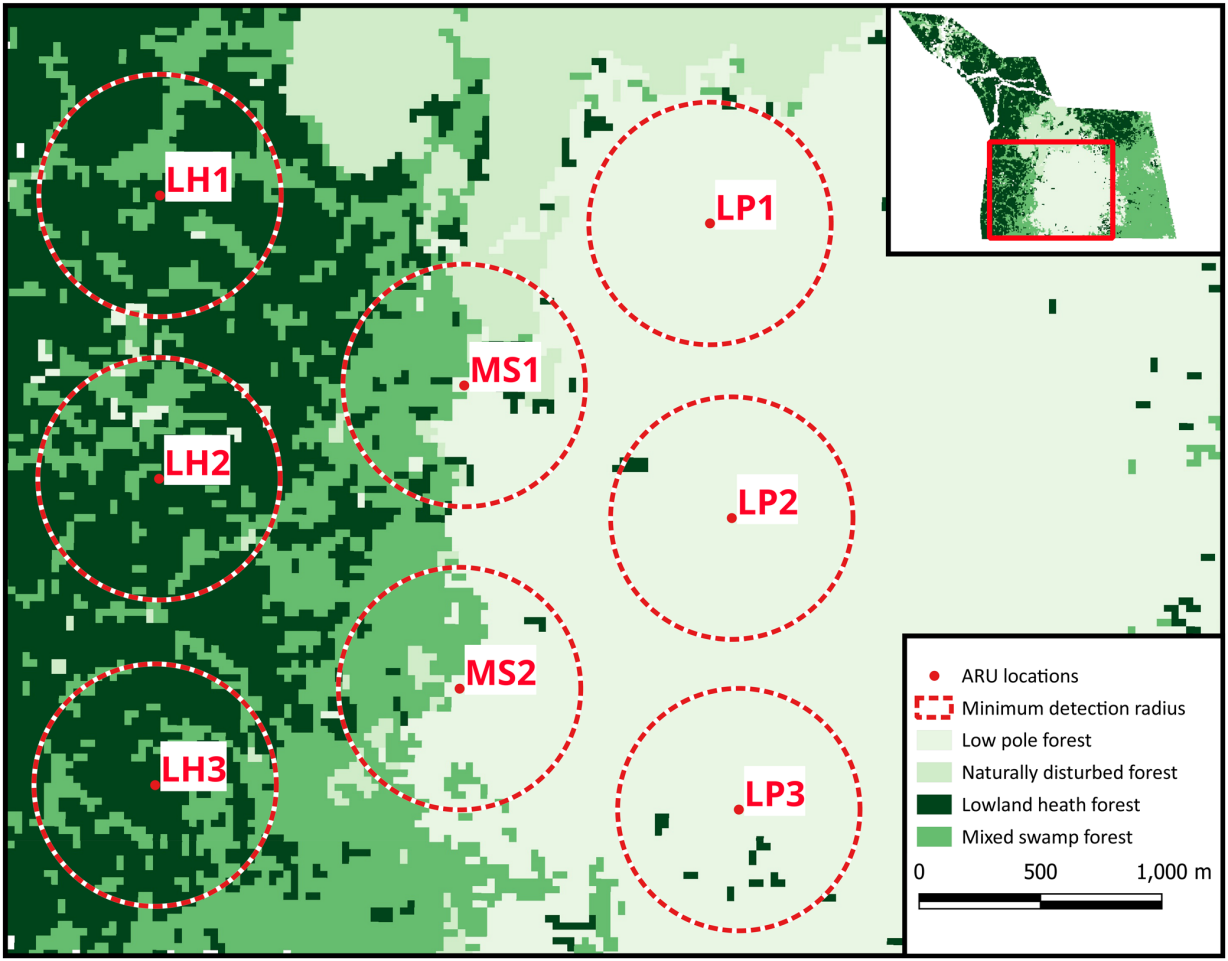
Map of the Mungku Baru Education and Research Forest showing the distribution of different habitat types over the survey area and the location of the autonomous recording units (ARUs; Buckley et al. 2018). The dashed red circles represent a predicted minimum 500 m detection radius around each ARU, based on playback experiments (Erb, unpublished data). The ARU labels correspond to the forest type where each device was deployed (LH—lowland heath, MS—mixed swamp, LP—low pole).

Eight autonomous recording units (ARUs; Song Meter SM4, Wildlife Acoustics, Maynard, Massachusetts) were deployed in the MBERF by W.M.E. and E.E. from July 2018 to December 2019. The ARUs were placed on trees, 5 m above the ground, in a dispersed grid with approximately 1200 m between devices. This placement ensured full coverage of the study area while aiming to minimise overlapping detections of ape calls between neighbouring ARUs. Playback experiments indicate that gibbon great calls can be detected in recordings from distances of 500 m or more (Erb, unpublished data). The ARU grid was designed to sample each of the habitats within the MBERF, wherein three were deployed in lowland heath, three in low pole, and two in mixed swamp habitats (Figure 2).

The ARUs were programmed to record daily from 4 am to 6 pm (local time, UTC +7) to capture the predawn and diurnal period of ape calling. These used default settings [sensitivity of −35 ± 4 dB (0 dB = 1 V/pa at 1 kHz), dynamic range of 14–100 dB sound pressure level (SPL) at 0 dB gain, microphone gain of 16 dB, and inbuilt preamplifier gain of 26 dB] and recorded on two channels with a sampling rate of 24 kHz. Audio was captured in 16‐bit waveform audio file format (WAV) and saved as 1‐h files. Memory cards and batteries were changed every 2 weeks. Due to logistical and technical difficulties, devices did not always record continuously throughout the full survey period. To ensure full coverage of gibbon duets, we selected files recorded between 04:00 and 10:00 and only included days with no missing data. The average number of survey days per recorder was 484 (range = 429–498 of a possible 535 days; Table S1). The resultant dataset contained 23,244 h of audio.

To detect *H. albibarbis* great calls, we applied the convolutional neural network (CNN) detector described in Owens et al. (2024). This was trained to recognise *H. albibarbis* great calls from spectrogram representations of audio recordings and was developed using Koogu (version 0.7.2), an open‐source deep learning framework for bioacoustic data (Madhusudhana 2023). The detector splits audio files into 28 s segments with a 27 s overlap and assigns each segment a confidence score (ranging from 0 to 1), with higher scores indicating greater confidence in the presence of a great call. We used a confidence score threshold of 0.78 to balance precision (the proportion of true positive predictions among all positive predictions) and recall (the proportion of true positive predictions among all actual positive instances), enabling reliable detection while minimising false positives (Owens et al. 2024). Neighbouring segments with scores above the threshold were grouped into detections (see Owens et al. 2024) to estimate the number of great calls per day at each ARU (*daily call rate*), and whether or not a great call was detected on a given day at each ARU (*daily call presence*). Together, these variables constitute daily calling activity, which served as the basis for subsequent analysis.

To estimate rainfall variables for our study site, we used the PERSIANN‐CDR V3 dataset, a high‐resolution (0.04° × 0.04°, 4 × 4 km) global satellite precipitation climate data record (Ashouri et al. 2015), accessed via the CHRS data portal (Center for Hydrometeorology and Remote Sensing (2014), University of California, Irvine). Daily rainfall accumulations were extracted for April 2018 to December 2019 from the grid cell centred at 1.64°S, 113.74°E, located approximately 3 km from the centrepoint of the ARU grid. All ARUs fell within or immediately adjacent to this single grid cell.

### 2.3 Detector Validation

To evaluate the precision of the automated detector across conditions, we visually assessed a subset of detections using Raven Pro 1.6 (K. Lisa Yang Center for Conservation Bioacoustics 2024). Specifically, we reviewed all detections from one randomly selected date per month between July 2018 and December 2019, restricted to dates when recordings were available from all ARUs (*n* = 864 sound files). This yielded 2923 detections in 326 sound files. Detections were annotated as true positives (TPs) if they overlapped with a great call or false positives (FPs) if they did not. We fitted a generalised linear model (GLM) with a quasibinomial distribution, using *precision* (Table 1) as the response variable. *Month*, *ARU* and *hour* (Table 1) were added as categorical predictor variables to assess whether these factors accounted for variation in precision. Nested models were compared using analysis of variance (ANOVA).

**TABLE 1:** Summary of response variables, predictor variables and random effects included in models for data analysis and detector validation.

	Description
**Data analysis**	
*Response variable*	
Daily call rate	Number of great calls detected per day at each ARU
Daily call presence	Binary variable indicating presence (1) or absence (0) of a great call at each ARU on a given day
*Predictor variable*	
Habitat	Habitat type in which the ARU was located
Sampling date	Date of each observation
Daily rainfall	Total daily rainfall (mm)
*Random effect*	
ARU	Unique ID of each recording unit
**Detector validation**	
*Response variable*	
Precision	Proportion of detections classified as true positives
Recall	Proportion of annotated calls successfully detected
*Predictor variable*	
Month	Month‐year of data collection
Hour	Recording hour
Call quality	Description of the clarity of great call annotations: ‘*clear*’, ‘*faint*’, or ‘*very faint*’ (see Owens et al. 2024)
ARU	Unique ID of each recording unit

To evaluate recall, we assessed detector performance relative to a manually annotated test dataset comprising 522 great calls in 90 sound files described in Owens et al. (2024). Great calls were annotated as TPs if they overlapped a model detection, and false negatives (FNs) if they did not. A GLM with a quasibinomial distribution was fitted with *recall* (Table 1) as the response variable. *Call quality* (Table 1), *month*, *ARU* and *hour* were included as categorical predictor variables to assess their effect on recall. Additionally, to test if the effect of *call quality* varied by *ARU*, *hour* and *month*, we tested models including interaction terms between *call quality* and each of these variables. Model selection was performed using ANOVA.

For the best‐fitting models for both precision and recall, we computed estimated marginal means using the R package ‘emmeans’ (Lenth 2025) to assess detector performance across predictor variables. Pairwise post hoc comparisons were conducted with Tukey adjustments for multiple testing. Confidence intervals for observed precision and recall were calculated from raw detection counts using Wilson's method to provide robust interval estimates. Finally, to assess whether variation in precision could be explained by the number of great calls for each condition, we tested for correlations between precision and the number of TPs for significant predictor variables using Spearman's rank correlation.

### 2.4 Data Analysis

To test whether daily calling activity varies among habitat types (*hypothesis 1*), and seasonally, with temporal trends differing among habitats (*hypothesis 2*), we fitted a generalised linear mixed model (GLMM) with a hurdle negative binomial (NB) distribution using the R package ‘glmmTMB’ (Brooks et al. 2017). The hurdle NB distribution was selected to account for excess days with zero detected calls and overdispersion in the count data (Bhaskar et al. 2023). This approach simultaneously addresses two aspects of daily calling activity: the binary component models whether calls were detected on a given day (*daily call presence*), while the count component models the number of calls (*daily call rate*) on days when calls were detected. *ARU* was included as a random effect in both model components to account for variation in daily calling activity among recording units within the same habitat, and to limit pseudo‐replication. We modelled temporal variation using a flexible smooth curve fitted to *sampling date* (Table 1), with the degree of flexibility selected by Bayesian Information Criterion, which identified df = 8 as the most parsimonious fit. We investigated the effects of *habitat*, *sampling date*, and their interaction on both *daily call rate* and *daily call presence*.

To test whether daily calling activity is positively associated with seasonal rainfall (*hypothesis 3*), we fitted a distributed lag non‐linear model (DLNM) implemented within the hurdle GLMM framework described above, using the R package ‘dlnm’ (Gasparrini 2011). This enabled us to simultaneously estimate the shape of the rainfall‐response relationship and how the effect changes across lag times, capturing both short‐ and long‐term effects of rainfall on *daily call rate* and *daily call presence*. Rainfall on the day of observation was excluded, as it could encompass rainfall occurring after the morning song bout. A cross‐basis matrix was constructed from *daily rainfall* (Table 1) lagged 1–100 days prior to observation and parameterised using flexible smooth curves on both the rainfall dose dimension and the lag dimension, with degrees of freedom selected by BIC grid search (dose df = 2, lag df = 3). *ARU* was included as a random effect in both model components.

Nested model comparisons were conducted using ANOVA and model fit was evaluated using Akaike's Information Criterion (AIC) and BIC.

## 3 Results

### 3.1 Detector Validation

To assess potential factors influencing the precision of the automated detector, we fitted a GLM with a quasibinomial distribution, using *precision* as the response variable and *month, ARU* and *hour* as categorical predictor variables. Initial model fitting suggested that *month* improved overall model fit (*F* = 2.127, *p* < 0.01). However, examination of individual *month* coefficients revealed no significant effects for any specific month, with some months showing extremely large standard errors indicative of sparse data. Therefore, *month* was excluded from the final model, which included *ARU* and *hour* as predictors (Figure S1). Precision varied across ARUs, with LH3 showing the highest precision (0.962, 95% CI: 0.942–0.975) and LP3 the lowest (0.716, 95% CI: 0.648–0.775). Post hoc pairwise comparisons indicated that precision was significantly higher at LH3 compared to LP2 (*p* < 0.05) and LP3 (*p* < 0.01). No other pairwise differences among ARUs were statistically significant after adjustment. Precision also varied significantly by *hour*. The highest precision occurred at 6 am (0.982, 95% CI: 0.973–0.988) and the lowest at 4 am (0.238, 95% CI: 0.165–0.329). Pairwise comparisons showed that precision was significantly lower at 4 am than all other hours of the morning, except for 9 am (*p* = 0.328). Additionally, precision at 6 am was significantly higher than at 8 am (*p* < 0.001) and 9 am (*p* < 0.001). Precision was positively associated with the number of TPs for both *ARU* (*ρ* = 0.929, *p* < 0.01) and *hour* (*ρ* = 0.943, *p* < 0.05; Figure S2). This suggests that a lower precision under certain conditions reflects a scarcity of great calls rather than a systematic increase in false detections.

For recall, we also fitted a GLM with a quasibinomial distribution, this time using *recall* as the response variable, and *call quality*, *month*, *ARU* and *hour* as categorical predictor variables. The best‐fitting model included *call quality* as the sole predictor (Figure S3). We then tested interaction terms between *call quality* and the other predictor variables, with only the *call quality × month* interaction being significant (*F* = 2.15, *p* < 0.05). However, examination of individual interaction coefficients again revealed extremely large standard errors indicative of sparse data, so interactions were excluded. Recall varied by *call quality*, with the greatest recall observed for ‘clear’ calls (0.951, 95% CI: 0.920–0.971) and the lowest for ‘very faint’ calls (0.406, 95% CI: 0.332–0.485). All pairwise comparisons were significant, showing that recall decreased with *call quality*.

### 3.2 Detector Output

**FIGURE 3:**
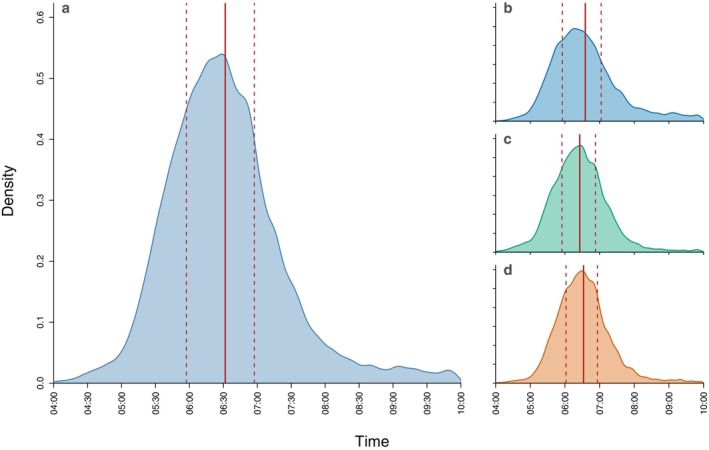
Density plot showing the temporal distribution of 83,956 great call detections between 04:00 and 10:00, shown for all habitats combined (a) and separately for lowland heath (b; *n* = 35,477), mixed swamp (c; *n* = 22,791), and low pole (d; *n* = 25,688). The solid red line indicates the mean detection time, and the dashed red lines indicate the interquartile range.

Over the survey period, 83,956 *H. albibarbis* great calls were identified by the automated detector across all ARU devices, with a mean of 10,545 great calls per ARU (range: 6463 at LP3–14,915 at LH3, SD = 2756). Calls were detected on a mean of 86.3% of survey days across ARUs (range: 78.1% at LP3–94.3% at LH3, SD = 5.1%). The mean *daily call rate* per ARU, on days when calls were detected, ranged from 16.6 (LP3) to 33.3 (LH3), with an overall mean of 24.9 (SD = 5.1) calls per day. Great calls were detected across the entire 04:00–10:00 period, with half of all detected great calls occurring between 05:57 and 06:57, and a mean time of 06:32 (Figure 3). This pattern was broadly consistent across habitat types, with mean detection times ranging from 06:26 in mixed swamp to 06:35 in lowland heath.

### 3.3 Habitat Differences in Calling Activity

To test whether daily calling activity differs among habitat types (*hypothesis 1*), we added *habitat* to the *sampling date*‐only model, which resulted in a significant improvement in model fit (χ^2^ = 11.157, df = 4, *p* < 0.05) and the lowest AIC among candidate models, though the *sampling date*‐only model had a lower BIC (Table S2). The model showed a significant negative effect of low pole on daily calling activity relative to lowland heath (*daily call rate*: *z* = −2.346, *p* < 0.05; *daily call presence*: *z* = −2.999, *p* < 0.01), whereas calling activity in mixed swamp did not differ significantly from lowland heath (*daily call rate*: *z* = 0.043, *p* = 0.965; *daily call presence*: *z* = −1.798, *p* = 0.072). These results support a significant effect of habitat on daily calling activity (*hypothesis 1*), with lower calling activity associated with lower and more discontinuous canopy cover, though this conclusion is sensitive to the choice of information criterion.

### 3.4 Temporal Variation in Calling Activity

**FIGURE 4:**
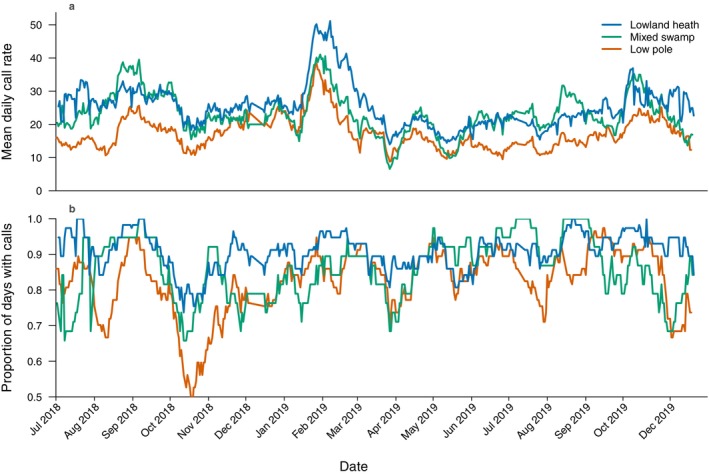
Mean daily call rate (number of great calls detected per day; a) and the proportion of days with calls (daily call presence; b) across three habitat types. Values are smoothed using a 19‐day centred rolling mean across ARUs within each habitat (lowland heath *n* = 3, mixed swamp *n* = 2, low pole *n* = 3).

To test whether daily calling activity varies seasonally, with temporal trends differing among habitats (*hypothesis 2*), we examined the effect of *sampling date* and its interaction with *habitat*. Including *sampling date* as a predictor significantly improved model fit compared to a model with *habitat* alone (χ^2^ = 209.900, df = 16, *p* < 0.001) and lowered both AIC and BIC (Table S2). There was significant temporal variation in daily calling activity, with distinct peaks and troughs over the survey period (Figure 4). Further, including an interaction between *habitat* and *sampling date* did not improve model fit compared to a model with only the main effects (χ^2^ = 45.220, df = 32, *p* = 0.061). These results partially support *hypothesis 2*, showing significant temporal variation in daily calling activity, though observed temporal trends did not differ significantly across habitat types.

### 3.5 Calling Activity Reflects Prior Rainfall

**FIGURE 5:**
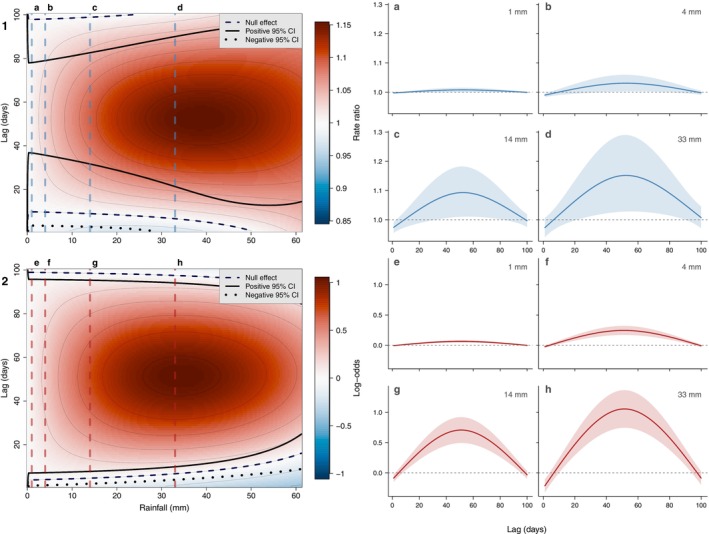
Association between daily calling activity and prior daily rainfall accumulations, estimated using a distributed lag non‐linear model (DLNM). Panels 1 and 2 show the rainfall × lag‐response surface for daily call rate and daily call presence, respectively, indicating where the effect is null and the positive and negative 95% confidence interval boundaries. Vertical dashed lines indicate the rainfall doses at which lag‐response curves are shown in panels (a–d) (daily call rate) and (e–h) (daily call presence), at the 25th (1 mm), 50th (4 mm), 75th (14 mm) and 90th (33 mm) percentiles of non‐zero daily rainfall.

To test *hypothesis 3*, we examined the relationship between daily calling activity and prior rainfall. Including the cross‐basis matrix of *daily rainfall* as a predictor significantly improved model fit compared to the *habitat + sampling date* model (χ^2^ = 109.400, df = 12, *p* < 0.001) and lowered both AIC and BIC (Table S3), indicating a significant association between prior rainfall and daily calling activity. The DLNM surface revealed a positive association between daily calling activity and rainfall in the preceding weeks to months, with effect sizes increasing with rainfall dose (Figure 5). At the 90th percentile of non‐zero *daily rainfall* (33 mm), *daily call rate* was significantly higher 22–89 days later, peaking at 52 days after rainfall (RR = 1.152, 95% CI: 1.028–1.290 at 33 mm; Table S4). The equivalent effect on *daily call presence* was stronger and detectable at lower rainfall doses, with a significantly higher probability of calling 10–94 days later, peaking at 51 days after rainfall (log‐odds = 1.057, 95% CI: 0.746–1.368 at 33 mm). Notably, the lag at which the effect peaked was consistent across all rainfall percentiles examined, for both *daily call rate* (52 days) and *daily call presence* (51 days), though the effect sizes were substantially smaller at lower rainfall doses.

A short‐term suppressive effect of rainfall on daily calling activity was also evident. Rainfall 1 day prior to observation had a significant negative effect on *daily call presence* at all rainfall doses (e.g., log‐odds = −0.215, 95% CI: −0.322 to −0.108 at 33 mm; Table S4), while the effect on *daily call rate* was weaker and non‐significant at higher rainfall doses (RR = 0.973, 95% CI: 0.941–1.007 at 33 mm), becoming significant only at lower rainfall doses (e.g., RR = 0.974, 95% CI: 0.955–0.993 at 14 mm).

**FIGURE 6:**
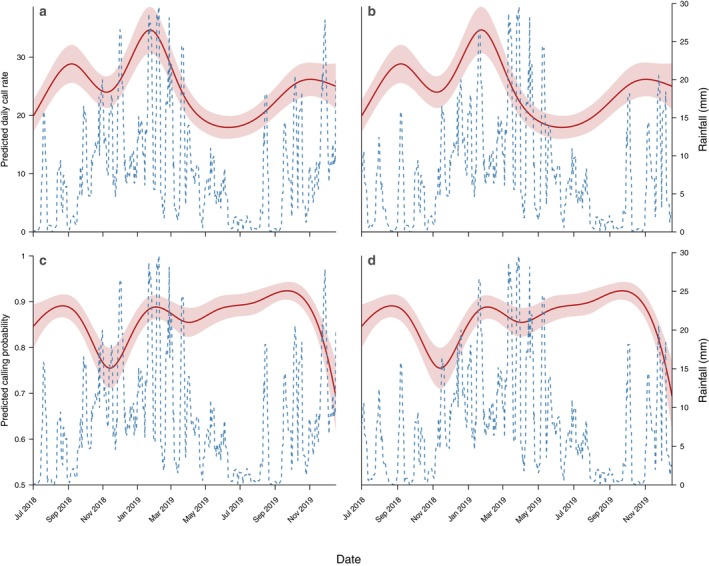
Model‐predicted daily calling activity and prior rainfall across two lag windows. Panels (a, b) show predicted daily call rate; panels (c, d) show predicted calling probability. In all panels, the red line shows model‐predicted calling activity (habitat + sampling date model) with shaded 95% confidence intervals. The blue dashed line shows mean daily rainfall over the preceding 7 days (a, c) and over days 50–56 prior to observation (b, d).

These findings support *hypothesis 3*, demonstrating a positive association between seasonal rainfall and daily calling activity, despite a short‐term suppressive effect of rainfall on calling. However, this positive association was not uniform across the survey period, with a notable deviation from March to May 2019, when daily calling activity declined markedly despite elevated rainfall in the preceding ~50 days (Figure 6).

## 4 Discussion

Studying the ecology of elusive species inhabiting tropical forests remains challenging, such as for *H. albibarbis* , where large parts of the species' range remain understudied. To address this, we deployed eight ARUs across a mosaic lowland forest landscape over 18 months to examine differences in calling patterns among forest types and assess the effects of rainfall on vocal behaviour. Our results highlight spatiotemporal variability in *H. albibarbis* daily calling activity, associated with rainfall patterns, suggesting an influence of seasonal fluctuations in resource availability.

We observed significant differences in daily calling activity between habitats, with a lower occurrence of great calls in low pole compared to lowland heath. This aligns with our expectations, as low pole has a lower, discontinuous canopy cover, which has been associated with lower gibbon densities (Hamard et al. 2010), and lower densities are in turn associated with less calling activity (Brockelman and Srikosamatara 1993; Yin et al. 2016). Our findings are broadly consistent with previous observations, where low pole was presumed to support a low to near‐zero group density based on infrequent duetting and sparse gibbon sightings in this habitat (Buckley et al. 2006; Cheyne et al. 2008).

However, our model results for *habitat* should be interpreted with caution, with BIC favouring the simpler *sampling date*‐only model, indicative of limited statistical power with only 2–3 replicates per habitat. Direct habitat comparisons are further complicated by variation in great call propagation distance among forest types, with higher propagation distances observed in lowland heath compared to mixed swamp and low pole (Erb, unpublished data), potentially inflating differences in detected call rates. Further, ARU placement relative to gibbon home range boundaries introduces additional uncertainty, as ARUs located centrally within a group's home range may detect fewer calls than those positioned at the boundary between multiple groups, where calls from a greater number of individuals may be recorded. Finally, consistent differences in the rate of detected calls across ARUs may reflect variation in individual or group‐level calling behaviour that does not necessarily correspond to group density (Clink et al. 2020; Ma et al. 2022). Despite these limitations, the detection of a significant habitat effect is nonetheless informative, and applying acoustic localisation methods such as the time difference of arrival technique (Lellouch et al. 2025) would allow calls to be attributed to distinct groups in future studies, enabling more robust estimates of relative gibbon population densities across habitat types using PAM.

We observed significant temporal variation in daily calling activity, with distinct peaks and troughs over the survey period. This was associated with seasonal rainfall, with the DLNM revealing significant positive effects of rainfall on both the likelihood of calling and daily call rates, peaking ~50 days after rainfall, and increasing with rainfall dose. Daily call rates were highest during the rainy season, particularly in February 2019, despite the well‐documented negative short‐term effects of rainfall on gibbon calling behaviour (Brockelman and Srikosamatara 1993; Cheyne 2008; Clink et al. 2020; Mitani 1985). Given that the rainy season is associated with peaks in animal‐dispersed fruit abundance (van Schaik and Pfannes 2005), and that gibbons call more during periods of higher food availability (Cowlishaw 1996), our findings are consistent with the hypothesis that resource availability is an important driver of gibbon singing behaviour. The lag effect captured by the DLNM could reflect a phenological fruiting response to seasonal rainfall, whereby fruit availability peaks in the rainy season either as a direct response to rainfall or following drought‐induced flowering during the preceding dry season (van Schaik and Pfannes 2005; Brearley et al. 2007; Dunham et al. 2018; Kurten et al. 2018). Notably, temporal variation in daily calling activity was broadly consistent across habitats, suggesting that fruiting responses to rainfall across the landscape are similar, or that the key food species driving temporal variation in calling activity are present in all three forest types. Concurrent monitoring of fruiting phenology alongside gibbon calling activity, over a longer survey period spanning multiple years with contrasting rainfall regimes, would help clarify whether the observed lag effect reflects a consistent seasonal fruiting response or is driven by a specific inter‐annual phenological event. This distinction cannot be resolved from our 18‐month dataset alone, but could ultimately allow gibbon vocal patterns to serve as a broader indicator of habitat productivity over time.

Our results also confirm the significant negative effects of rainfall on daily calling activity in the short‐term, with rainfall 1 day prior to observation reducing the probability of calling across all rainfall doses. This may reflect increased energetic costs of overnight thermoregulation associated with adverse weather, reducing motivation to call the following morning (Cheyne 2008; Clink et al. 2020). However, the suppressive effect of rainfall on daily call rates was only significant at lower rainfall doses. This is likely because higher rainfall doses decrease the probability of calling at all, leaving fewer observations for the call rate component of the hurdle model and reducing statistical power to detect a significant effect. The suppressive effect of rainfall appeared to periodically override the lagged positive association of rainfall with calling activity, particularly towards the end of the rainy season in March to May 2019 despite heavy rainfall in the preceding ~50 days. During the dry season, calling probability remained high while call rates were relatively low, particularly from May to September 2019. This is consistent with reduced rainfall allowing for more days with calling, while lower resource availability limited call rates. Together, our results reveal opposing short‐ and long‐term effects of rainfall on gibbon calling activity, aligning with temporal patterns of singing behaviour observed throughout the survey period.

The occurrence of the El Niño weather event in 2019 may have further influenced observed patterns of calling activity. The associated drought likely lowered resource availability during the dry season (van Schaik and Pfannes 2005), potentially contributing to the low call rates observed from May to September 2019. This event also triggered forest fires across Borneo, which blanketed large areas in a smoke haze and increased levels of harmful pollutants such as PM_2.5_ and carbon monoxide, particularly from September to November 2019 (Yokelson et al. 2022). Wildfire smoke has been shown to negatively affect *H. albibarbis* singing behaviour, reducing both the number of singing days per month and the length of song bouts, potentially due to respiratory stress (Cheyne 2008). Similarly, Erb et al. (2023) found that Bornean orangutans ( *Pongo pygmaeus wurmbii* ) perform fewer long calls per day when exposed to increased air pollution from wildfire smoke. Although it remains unclear whether wildfire smoke directly influenced our results, our study highlights the importance of accounting for underlying variation in vocal patterns when examining the additive effects of wildfire smoke on animal communication.

The performance of the deep learning detector varied significantly across ARUs, times of day, and the quality of the target calls. While the lower precision for LP3 may have contributed to a slightly inflated daily call rate in low pole due to a higher number of false positive detections, this ARU recorded the fewest calls overall. Similarly, although precision was lowest for files recorded at 4 am, this hour accounted for a relatively small proportion of total detections. The markedly low precision during this hour likely reflects the presence of male solo calls preceding the duet (Clink et al. 2020), some of which were misclassified as great calls. Furthermore, precision for both *ARU* and *hour* was positively correlated with the number of true positives present, suggesting a relatively stable false positive rate, which would result in lower precision where great calls are scarce, rather than a systematic increase in false detections. As expected, recall varied with *call quality*, since ‘clear’ calls are more likely to originate closer to the ARU than ‘very faint’ calls. Further investigation into the relationship between detection probability and distance to the call source would help to determine effective ARU sampling areas. Together, these patterns suggest that observed temporal variation in daily calling activity is more likely attributable to ecological processes than to fluctuations in detector performance. However, caution is warranted when interpreting daily call rates during off‐peak calling hours and in low‐density populations. Overall, our validation process highlights the importance of comprehensive performance assessment when interpreting the output of automated detectors in ecological applications, with particular attention to generating representative test datasets when applying detection across different locations, habitats, and time periods.

This study highlights the advantages of applying deep learning detectors to acoustic data when studying species' vocal behaviour. Although frequent maintenance of ARUs was required, conducting manual acoustic surveys at eight locations simultaneously over an equivalent time period would be logistically infeasible. Furthermore, the 23,244‐h dataset was analysed by our automated detector in only ~123 h. Unlike manual acoustic surveys, our approach allows for continuous monitoring without the presence of human observers, which could influence vocal behaviour (Reisland and Lambert 2016). The scalability of this method from single‐site to landscape‐level monitoring provides opportunities to study species' distributions and responses to environmental or anthropogenic factors across habitats and over extended time periods. This approach is particularly applicable to loud‐calling primate species with fixed territories, such as other gibbon species or howler monkeys (Pérez‐Granados and Schuchmann 2021), but can be extended more broadly to track temporal variation in vocal activity across a range of vocally conspicuous taxa, including birds, amphibians, and insects. Our findings suggest that lowland heath forests support healthy gibbon populations, as evidenced by the relatively high call rates recorded in this habitat. Although widespread, lowland heath forests have often been overlooked in conservation planning due to misconceptions about their biodiversity and ecological productivity (Anirudh et al. 2025). Our results challenge this perception by highlighting the importance of lowland heath for endangered *H. albibarbis* , thereby strengthening the case for protecting this habitat type in future conservation strategies.

## Author Contributions

**Alasdair F. Owens:** conceptualization (lead), formal analysis (lead), methodology (lead), project administration (lead), writing – original draft (lead), writing – review and editing (lead). **Erik Estrada:** conceptualization (equal), data curation (equal), writing – review and editing (equal). **Kimberley J. Hockings:** conceptualization (equal), supervision (equal), writing – review and editing (equal). **Muhammed Ali Imron:** conceptualization (equal), project administration (equal), writing – review and editing (equal). **Mariaty:** conceptualization (equal), project administration (equal), writing – review and editing (equal). **Manmohan D. Sharma:** formal analysis (equal), resources (supporting), supervision (equal), writing – review and editing (equal). **Siti Maimunah:** conceptualization (equal), project administration (equal), writing – review and editing (equal). **Tommy J. Travers‐Cook:** conceptualization (equal), formal analysis (supporting), methodology (supporting), writing – review and editing (equal). **Frank J. F. Van Veen:** conceptualization (equal), formal analysis (supporting), methodology (supporting), project administration (supporting), resources (supporting), supervision (equal), writing – review and editing (equal). **Wendy M. Erb:** conceptualization (equal), data curation (lead), resources (supporting), supervision (equal), writing – review and editing (equal).

## Funding

This work was supported by the International Primatological Society. American Association of Biological Anthropologists. Margot Marsh Biodiversity Foundation. Natural Environment Research Council, NE/S007504/1. American Institute for Indonesian Studies. British Academy, VF1\100400.

## Ethics Statement

Ethical approval was provided by the University of Exeter (Application ID 1845574), BRIN (Application No. 22022023000026), and the Institutional Animal Care and Use Committee of Rutgers, the State University of New Jersey Protocol No. PROTO201800073.

## Conflicts of Interest

The authors declare no conflicts of interest.

## Supporting information


**Table S1:** The number of survey days for each ARU per month.
**Table S2:** Model fit comparisons for hypotheses 1 and 2. A hurdle negative binomial model with a random effect of recording unit was fitted to test the effects of habitat, sampling date, and their interaction on daily calling activity.
**Table S3:** Model fit comparisons for hypothesis 3. A hurdle negative binomial model with a random effect of recording unit was fitted to test the effects of prior daily rainfall on daily calling activity. Daily rainfall was modelled as a distributed lag non‐linear model (DLNM) cross‐basis matrix, and compared to a null model including habitat and sampling date‐only.
**Table S4:** Lag‐response summaries from a distributed lag non‐linear model (DLNM) at the 25th (1 mm), 50th (4 mm), 75th (14 mm), and 90th (33 mm) percentiles of non‐zero daily rainfall lagged 1–100 days before observation, showing the effect of lagged rainfall on daily calling activity. The table shows the number of days before observation where significant effects peaked, the effect size and 95% confidence intervals (CIs) for the corresponding day, as well as the range of days before observation where daily rainfall had significant positive and negative effects on calling activity, respectively. †Effect size is expressed as a rate ratio (RR) for daily call rate and as log‐odds for daily call presence.
**Figure S1:** Precision of the automated detector by ARU (a) and hour (b) with 95% Wilson confidence intervals.
**Figure S2:** Precision of the automated detector by ARU (a) and hour (b) relative to the total number of true positives.
**Figure S3:** Recall of the automated detector by call quality with 95% Wilson confidence intervals.

## Data Availability

All data and scripts necessary to replicate the results are listed publicly available using the DOI: https://doi.org/10.5061/dryad.k98sf7mm5.
