# Supplementary material for: Passive Acoustic Monitoring and Deep Learning Reveal a Lag From Rainfall to Gibbon Song Across a Mosaic Forest Landscape
Source: Ecol Evol. 2026 May 27;16(6):e73717. doi: 10.1002/ece3.73717 (PMC13239700; doi:10.1002/ece3.73717)
Supplement: Supplementary file 1 — Table S1: The number of survey days for each ARU per month. Table S2: Model fit comparisons for hypotheses 1 and 2. A hurdle negative binomial model with a random effect of recording unit was fitted to test the effects of habitat, sampling date, and their interaction on daily calling activity. Table S3: Model fit comparisons for hypothesis 3. A hurdle negative binomial model with a random effect of recording unit was fitted to test the effects of prior daily rainfall on daily calling activity. Daily rainfall was modelled as a distributed lag non‐linear model (DLNM) cross‐basis matrix, and compared to a null model including habitat and sampling date‐only. Table S4: Lag‐response summaries from a distributed lag non‐linear model (DLNM) at the 25th (1 mm), 50th (4 mm), 75th (14 mm), and 90th (33 mm) percentiles of non‐zero daily rainfall lagged 1–100 days before observation, showing the effect of lagged rainfall on daily calling activity. The table shows the number of days before observation where significant effects peaked, the effect size and 95% confidence intervals (CIs) for the corresponding day, as well as the range of days before observation where daily rainfall had significant positive and negative effects on calling activity, respectively. †Effect size is expressed as a rate ratio (RR) for daily call rate and as log‐odds for daily call presence. Figure S1: Precision of the automated detector by ARU (a) and hour (b) with 95% Wilson confidence intervals. Figure S2: Precision of the automated detector by ARU (a) and hour (b) relative to the total number of true positives. Figure S3: Recall of the automated detector by call quality with 95% Wilson confidence intervals. [file ECE3-16-e73717-s001.docx]

# Appendix S1

| ARU | Jul-18 | Aug-18 | Sep-18 | Oct-18 | Nov-18 | Dec-18 | Jan-19 | Feb-19 | Mar-19 | Apr-19 | May-19 | Jun-19 | Jul-19 | Aug-19 | Sep-19 | Oct-19 | Nov-19 | Dec-19 | Total |
| --- | --- | --- | --- | --- | --- | --- | --- | --- | --- | --- | --- | --- | --- | --- | --- | --- | --- | --- | --- |
| LH1 | 15 | 20 | 30 | 31 | 23 | 29 | 25 | 28 | 31 | 30 | 31 | 30 | 31 | 25 | 30 | 31 | 30 | 29 | 498 |
| LH2 | 1 | 9 | 30 | 22 | 30 | 27 | 24 | 28 | 31 | 29 | 31 | 30 | 31 | 26 | 28 | 18 | 28 | 8 | 429 |
| LH3 | 12 | 30 | 30 | 31 | 30 | 26 | 23 | 27 | 31 | 30 | 31 | 28 | 30 | 26 | 25 | 26 | 22 | 23 | 475 |
| MS1 | 16 | 20 | 30 | 31 | 30 | 27 | 24 | 28 | 31 | 30 | 31 | 30 | 24 | 26 | 30 | 28 | 30 | 24 | 490 |
| MS2 | 14 | 24 | 30 | 31 | 30 | 24 | 23 | 28 | 31 | 30 | 31 | 30 | 30 | 26 | 30 | 23 | 30 | 28 | 493 |
| LP1 | 17 | 30 | 21 | 31 | 30 | 27 | 24 | 27 | 31 | 30 | 30 | 30 | 30 | 25 | 29 | 26 | 30 | 25 | 493 |
| LP2 | 14 | 31 | 30 | 31 | 30 | 27 | 24 | 27 | 31 | 30 | 31 | 30 | 31 | 26 | 30 | 23 | 30 | 22 | 498 |
| LP3 | 17 | 31 | 30 | 31 | 30 | 25 | 24 | 26 | 31 | 30 | 31 | 30 | 29 | 23 | 30 | 26 | 28 | 27 | 498 |

*Table S1. The number of survey days for each ARU per month.*

| Model | df | AIC | BIC | ΔAIC | ΔBIC |
| --- | --- | --- | --- | --- | --- |
| *habitat + sampling date* | 25 | 30819.93 | 30976.48 | 0.00 | 21.89 |
| *sampling date* | 21 | 30823.09 | 30954.59 | 3.16 | 0.00 |
| *habitat x month* | 57 | 30838.71 | 31195.65 | 18.78 | 241.06 |
| *habitat* | 9 | 30997.83 | 31054.19 | 177.90 | 99.60 |

*Table S3. Model fit comparisons for hypothesis 3. A hurdle negative binomial model with a random effect of recording unit was fitted to test the effects of prior daily rainfall on daily calling activity. Daily rainfall was modelled as a distributed lag non-linear model (DLNM) cross-basis matrix, and compared to a null model including habitat and sampling date only.*

*Table S2. Model fit comparisons for hypotheses 1 and 2. A hurdle negative binomial model with a random effect of recording unit was fitted to test the effects of habitat, sampling date, and their interaction on daily calling activity.*

| Model | df | AIC | BIC | ΔAIC | ΔBIC |
| --- | --- | --- | --- | --- | --- |
| *habitat + sampling date + daily rainfall* | 37 | 30734.53 | 30966.22 | 0.00 | 0.00 |
| *habitat + sampling date* | 25 | 30819.93 | 30976.48 | 85.4022 | 10.26 |

| Response variable | Rainfall (mm) | Peak lag (days) | Effect size*†* (RR/log-odds) | 95% CI | Sig. positive lags (days) | Sig. negative lags (days) |
| --- | --- | --- | --- | --- | --- | --- |
| *Daily call rate* | 1 | 52 | 1.008 | 1.001–1.015 | 37–78 | 1–3 |
|  | 4 | 52 | 1.031 | 1.003–1.059 | 36–79 | 1–3 |
|  | 14 | 52 | 1.093 | 1.010–1.182 | 32–82 | 1–2 |
|  | 33 | 52 | 1.152 | 1.028–1.290 | 22–89 | – |
| *Daily call presence* | 1 | 51 | 0.064 | 0.045–0.084 | 8–95 | 1 |
|  | 4 | 51 | 0.245 | 0.170–0.320 | 8–95 | 1 |
|  | 14 | 51 | 0.708 | 0.494–0.922 | 8–95 | 1–2 |
|  | 33 | 51 | 1.057 | 0.746–1.368 | 10–94 | 1–3 |

*Table S4. Lag-response summaries from a distributed lag non-linear model (DLNM) at the 25th (1 mm), 50th (4 mm), 75th (14 mm), and 90th (33 mm) percentiles of non-zero daily rainfall lagged 1–100 days before observation, showing the effect of lagged rainfall on daily calling activity. The table shows the number of days before observation where significant effects peaked, the effect size and 95% confidence intervals (CIs) for the corresponding day, as well as the range of days before observation where daily rainfall had significant positive and negative effects on calling activity, respectively. †Effect size is expressed as a rate ratio (RR) for daily call rate and as log-odds for daily call presence.*


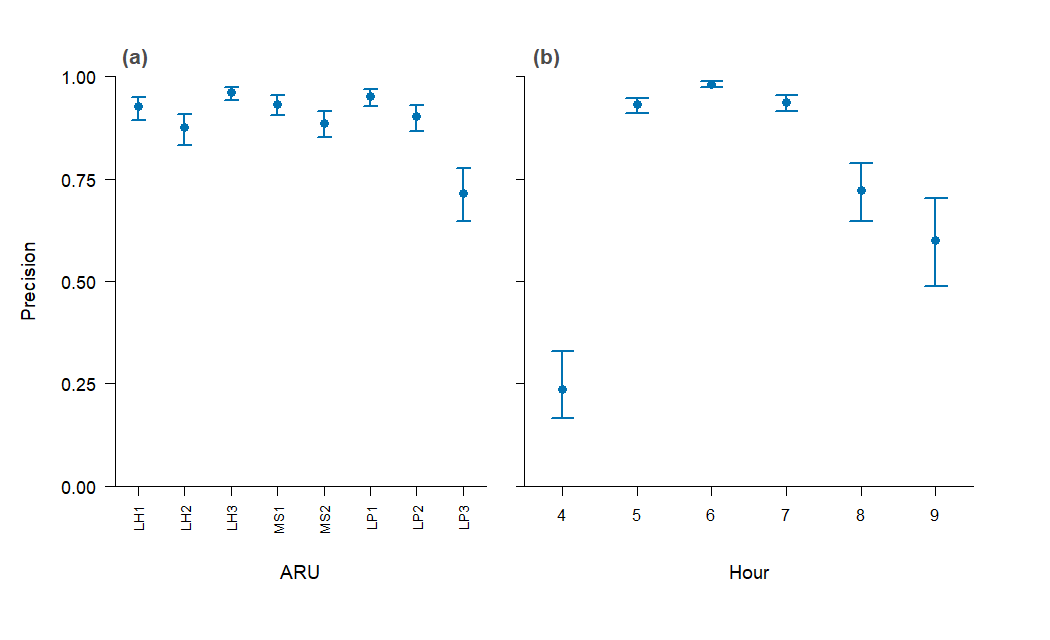


*Figure S1. Precision of the automated detector by ARU (a.) and hour (b.) with 95% Wilson confidence intervals.*


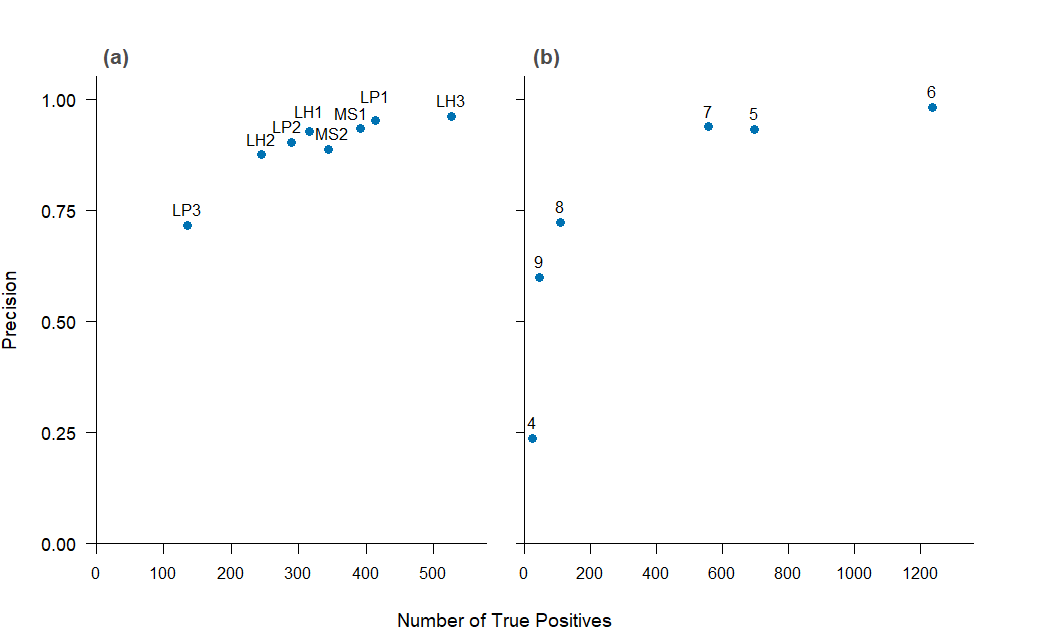


*Figure S2. Precision of the automated detector by ARU (a.) and hour (b.) relative to the total number of true positives.*


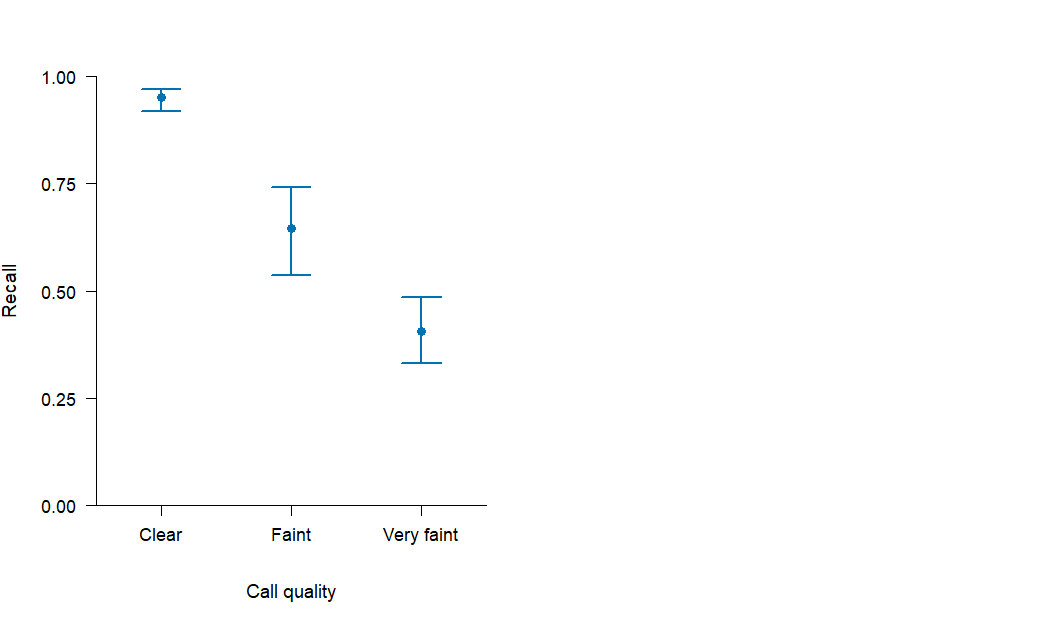


*Figure S3. Recall of the automated detector by call quality with 95% Wilson confidence intervals.*
